# Pollinator Power: Nutrition Security Benefits of an Ecosystem Service

**DOI:** 10.1289/ehp.123-A210

**Published:** 2015-07-31

**Authors:** Wendee Nicole

**Affiliations:** Wendee Nicole has written for *Discover*, *Scientific American*, and other publications.

The world has been abuzz with the dramatic losses of cultivated honey bees due to colony collapse disorder^1^ as well as declines of native pollinator species across the globe.^2^^,^^3^^,^^4^ Scientists have recently begun calculating the extent to which food crops depend on animal pollinators including bees, butterflies, and bats,^5^ with one study assigning an economic value to the “ecosystem service” provided by pollinators at approximately $167 billion.^6^ Even more recently, several other new studies have offered evidence that pollinators may also have a beneficial impact on nutrition security—the availability of essential macro- and micronutrients in the human diet.^7^^,^^8^^,^^9^

**Figure:**
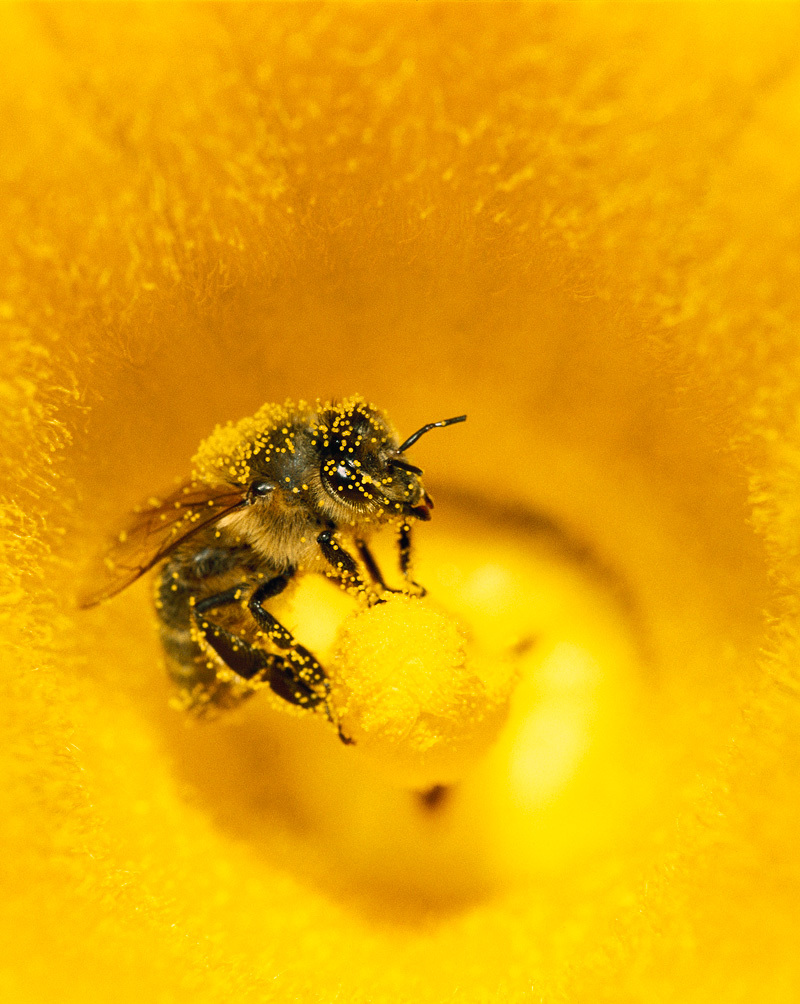
It’s well known that pollinators affect crop yields and thus market prices. New studies are showing they can affect the nutritional value of foods, too. © Konrad Wothe/Minden Pictures/Corbis

“It’s really well known that pollination changes the yields of crops and the economics of farming,” says Taylor Ricketts, director of the Gund Institute for Ecological Economics at the University of Vermont. It’s becoming better known, he says, that pollination also affects the nutritional value of foods.

Lack of the three macronutrients (fats, protein, and carbohydrates) and numerous essential micronutrients (vitamins and minerals) can cause specific nutrient-deficiency conditions as well as weaken the immune system, stunt development, and greatly increase mortality from other diseases.^10^^,^^11^ Already, about 795 million people worldwide chronically lack adequate calories and protein,^12^ and 2 billion suffer from micronutrient deficiencies (so-called hidden hunger).^11^ According to new estimates, a reduction in pollination services could worsen these problems in certain areas already struggling to overcome them.

## The Value of Bees

“Ecosystem services” are the seemingly free benefits provided by nature—provisions such as food and drinking water, life-sustaining processes such as water purification by wetland plants and nutrient cycling in soils, and more.^13^ The authors of the 2005 Millennium Ecosystem Assessment concluded that “any progress achieved in addressing the Millennium Development Goals of poverty and hunger eradication, improved health, and environmental sustainability is unlikely to be sustained if most of the ecosystem services on which humanity relies continue to be degraded.”^13^

Perhaps counterintuitively in some cases, human alteration of the natural world has coincided with improvements in many global health indices.^14^ At the same time, negative impacts of ecosystem changes also have become apparent and may become more so in the future.^14^ For many ecosystem services, there simply is not enough research to fully understand the associated human health impacts.

In one of the first attempts to assign value to pollination services, Alexandra-Maria Klein, an agroecologist at the University of Freiburg, and colleagues reviewed data on the extent to which global crop production relies on pollinators.^15^ For their analysis, Klein and colleagues selected 124 fruit, vegetable, and seed crops representing the top 99% of global food production, based on data from the Food and Agriculture Organization of the United Nations (FAO).

“We reviewed all the literature for each crop to find out how dependent it is on pollinators,” Klein says. “When you have the production value for each country, and you know how dependent each country is on pollinators, you can calculate what you lose [if pollinators disappear].”

Some degree of animal pollination was found to be necessary for 87 of the crops assessed, irrelevant for 28 others, and of unknown significance for the remaining 9. The crops that make up the greatest volume of global production (mainly cereal grains and sugarcane) rely on wind- and self-pollination. However, just over one-third of overall crop output comes from plants whose fruit, vegetable, or seed production increases with animal pollination.^15^

Klein followed this work with a study that estimated how pollinator declines might affect human nutrition.^7^ Her team collected FAO data on production of more than 150 crops gathered over the period 1997–2007, data from the U.S. Department of Agriculture on the macro- and micronutrient content of each crop, and Klein’s earlier data on crop pollinator dependence. Based on these data, they estimated that the majority of several micronutrients—vitamin A, vitamin C, and most carotenes and tocopherols—comes from crops that at least partially depend on animal pollinators (see table). For three micronutrients—vitamin A and the carotenes lycopene and β-cryptoxanthin—more than 40% was attributed solely to animal pollination.^7^

The team also estimated that 58% of calcium and 29% of iron comes from pollinator-dependent crops, with 9% and 6%, respectively, attributed solely to animal pollination.^7^ Although calcium and iron are absorbed more efficiently from meat and dairy sources, those foods are not available to all people due to high cost.^16^^,^^17^

**Figure:**
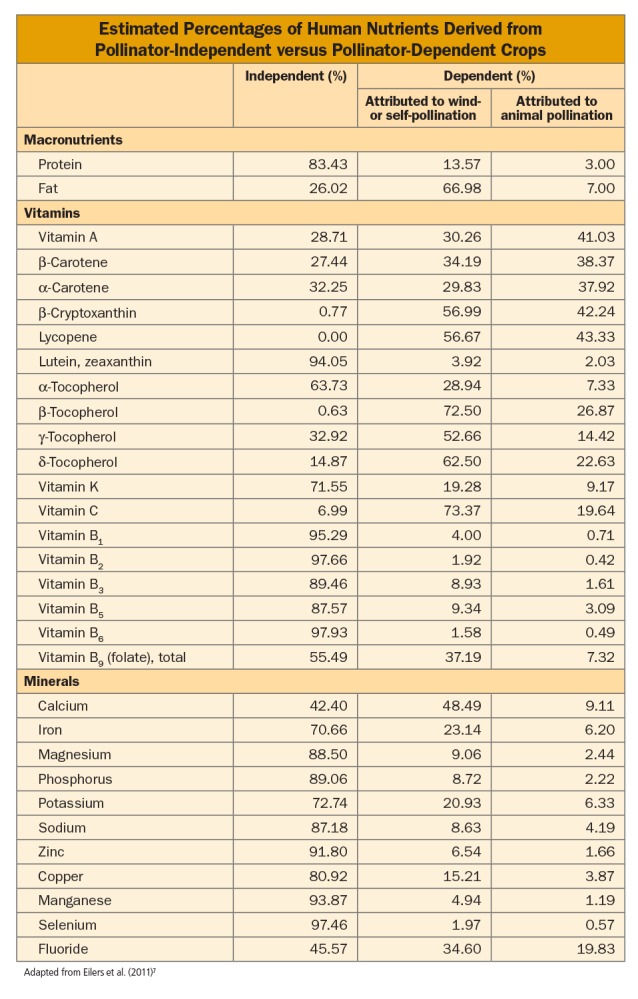


The authors concluded that jeopardizing animal-dependent pollination “could have a potentially drastic effect on human nutrition.”^7^ They acknowledged their findings were limited by the use of data generated in the United States, which may not reflect the nutrient content of the same foods grown in other countries. Barbara Herren, a program specialist in sustainable agriculture for the FAO, adds that the study also does not consider the traditional, local, and indigenous foods that many local communities depend on heavily, including wild foods gathered in the forest.

“Forests provide important dietary diversity to local populations, which depend on nontimber forest products to a much larger degree than is well understood,” Herren says. She points to recent research showing that children living in heavily forested areas of Africa tend to have more nutritious diets than children in areas with less tree cover.^18^ In addition, she says, “the demand for pollinator-dependent crops is increasing far faster in developing countries—where food and nutrition security are an issue—than in developed countries.”^19^

## Pollination and Nutrition Security

Animal pollinators appear to affect fruit condition, nutrient content, and hence market value in complex ways. One experiment found that bee-pollinated strawberries were redder, heavier, and firmer, and had reduced sugar–acid ratios—all leading to longer shelf life and higher market value—compared with wind- and self-pollinated fruits.^20^ Other studies have found animal pollination is associated with higher calcium content in apples,^21^ oil content in rapeseed,^22^ and sugar content in mandarin oranges.^23^

Klein and colleagues studied nutrient levels in almonds to determine whether they varied according to how the trees were pollinated.^24^ The researchers found lower levels of vitamin E but a higher ratio of oleic to linoleic acids in almonds from cross-pollinated trees compared with those from self-pollinated trees. There is evidence that almonds have cardioprotective qualities, which is attributed to their content of oleic acid, a monounsaturated fat.^25^ The researchers suggest that a higher ratio of oleic to linoleic acid (a polyunsaturated fat) would be desired by consumers looking for health benefits.^24^

In another study led by Klein, researchers found a strong relationship between pollination method and nut size.In an experimental orchard in the Sacramento Valley, they found that self-pollinated almond trees produced fewer and heavier nuts compared with hand-pollinated ones, with bee-pollinated almonds intermediate in size. The following season, the researchers gathered various sizes of nuts under normal orchard conditions, without experimental intervention, and found no association between nut weight and levels of nutrients per unit weight.^26^

“We thought maybe [the nutrient differences] are an indirect effect of, and triggered by, the size of the nut,” says Klein. “[But] we didn’t find a difference between size, so it needs to be related to pollinators.”

To take the emerging field a step further, Klein next joined forces with scientists from Stanford University’s Woods Institute for the Environment and the University of Minnesota’s Institute on the Environment through the Natural Capital Project, a group that maps and valuates ecosystem services. They wanted to identify regions where agriculture overall, as well as production of specific nutrients, depends most critically on pollination services. This information could help policy makers prioritize areas for pollinator conservation.

Using spatial data on the yield of 115 food crops around the world plus Klein’s data on crop pollinator dependence, they created maps depicting hot spots where production of heavily pollinator-dependent crops overlaps with deficiency in various micronutrients.^8^ “We ranked all nations by the extent of their pollination dependence for different micronutrients,” explains lead author Rebecca Chaplin-Kramer, a research associate for the Natural Capital Project. The team focused on three of the micronutrients most important for global health: vitamin A, iron, and folate. Vitamin A deficiency causes 800,000 deaths annually, doubling the mortality of several other diseases and quadrupling the rate of maternal mortality during childbirth.^27^ Iron deficiency is one of the world’s most common micronutrient deficiencies, causing preventable anemia, susceptibility to infection, and cognitive impairment.^28^ Folate is important in preventing neural tube defects in the developing fetus.^29^

Of these three micronutrients, vitamin A was estimated to be the most pollinator-dependent, approaching 50% in Thailand and scattered areas in India, Australia, Mexico, the United States, and other countries. Folate and iron reached a maximum of 12–15% pollinator dependence in parts of Asia, Mexico, Africa, and Brazil.^8^

The authors also mapped crop pollinator dependence against regions suffering from deficiencies of these essential micronutrients. They found that pollinator-dependent hot spots (where micronutrient production was more than 30% pollinator-dependent for vitamin A and more than 15% pollinator-dependent for iron) were three times more likely to occur in regions identified by the World Health Organization as at risk for vitamin A deficiency and iron-deficiency anemia (data to map folate deficiencies were unavailable).^8^

Chaplin-Kramer and colleagues also compared demand for the three micronutrients against pollinator-dependent supply. They estimated that pollinator-dependent production provides 13 times more folate and 5 times more vitamin A than the global population needs to meet daily intake recommendations, but only one-third of the iron needed.^8^

**Figure:**
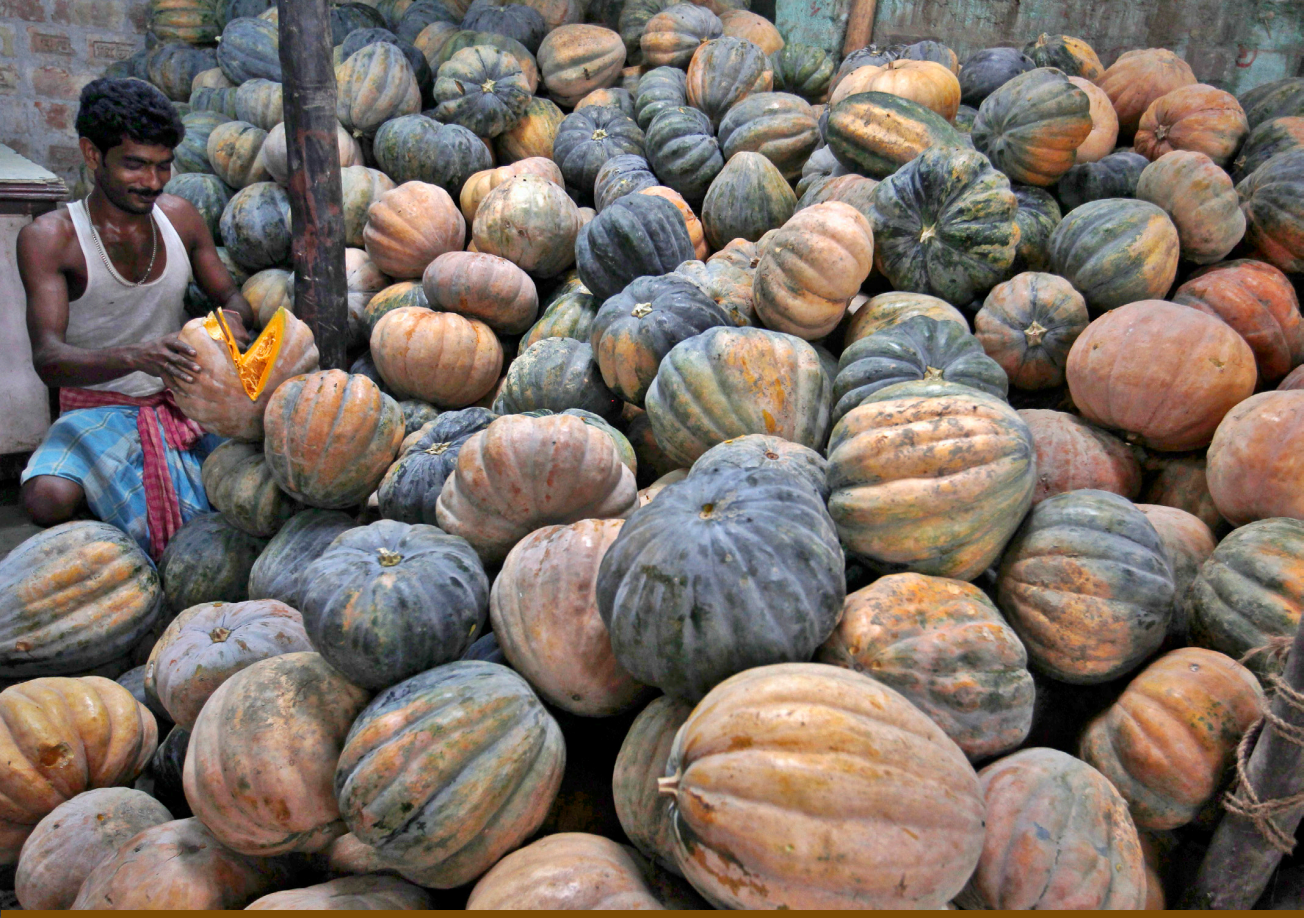
A Kolkata market vendor opens a pumpkin to check its quality. Pumpkins are a rich source of vitamin A, one of the most highly pollinator-dependent micronutrients. Studies of vitamin A and other important nutrients suggest that pollinator declines would affect different areas in different ways, depending on people’s current nutritional status, whether their usual sources of various nutrients depend on animal pollination, and the availability of nonpollinator-dependent alternative foods. © Rupak De Chowdhuri/Reuters/Corbis

But this supply–demand mismatch varies regionally, and production of vitamin A may be more limited in certain places. In parts of Southeast Asia, for example, Chaplin-Kramer says pollinator-dependent crops produce only 48% of the local demand. “That means there is already not enough vitamin A being produced [by crops] locally in Southeast Asia for people to reach their nutritional requirements,” Chaplin-Kramer says, “but in Central America [pollinator-dependent crops] produce way more than people could locally consume.” Although global trade can supplement local production, the fact that many of these countries are already malnourished suggests that the excess supply of micronutrients at a global level is irrelevant to the nutritional needs in many places.

Chaplin-Kramer says the findings have implications for both policy and science. “For policy, this demonstrates why the public health community should think more broadly about ecosystem services that may support or lead to further risk in nutritional health,” she says. The study also raises concern over whether continued pollinator losses will increase malnourishment and hidden hunger in regions where these conditions overlap with pollinator dependence.

“We need to be studying pollination in different places, based on where people may need it the most,” says Chaplin-Kramer. Such regions could be targeted for further studies, in contrast to the somewhat random nature of ecosystem services research up to this point.^14^

## Incorporating Diet

The next step is for investigators to examine how people in developing nations actually get their nutrition and whether that might change if pollinators were to disappear. In 2015 Ricketts, along with project lead Alicia Ellis of the University of Vermont and coauthor Samuel Myers of Harvard Medical School, reported findings from one such study.^9^ The study is one of the first projects of HEAL (Health & Ecosystems: Analysis of Linkages), a consortium of research institutions established to quantify the links between conservation, ecosystems, and human health.

“There’s a lot of talk about this, and some case studies, but we’re trying to systematically relate ecosystem change to health outcomes,” Ricketts says. “The focus of HEAL is to be as quantitative and clear and rigorous as we can.”

The researchers estimated how complete removal of pollinators—an unlikely scenario—would affect access to micronutrients of widespread health importance, namely, vitamin A, folate, and iron (as in the work by Chaplin-Kramer) as well as calcium and zinc. Calcium plays a key role in neuromuscular and skeletal development and function, while zinc is essential in numerous biochemical functions throughout the body.^29^ They used diet surveys from Uganda, Mozambique, Bangladesh, and Zambia to estimate levels of these micronutrients consumed in local diets. They analyzed nutrient intake for various groups but focused on children aged 1–3 years, because these nutrients are particularly important for growth and development.

Based on their analysis, the researchers predicted that pollinator loss would likely affect human health in highly variable ways, depending on local dietary preferences, the availability of alternatives to pollinator-dependent foods, and the state of people’s current nutrition.^9^ In Zambia, for instance, almost everybody studied was well nourished in vitamin A, to the point that individuals could absorb a loss of pollinator-dependent sources of this nutrient. In Bangladesh people were malnourished, and they had not been consuming pollinator-dependent foods high in vitamin A, so a reduction in pollinators likely would not change their nutritional status, either.

By contrast, in Uganda and Mozambique, many people were on the threshold of vitamin A deficiency. Ellis says these individuals had been getting much of their vitamin A from pollinator-dependent foods, and in those populations, the loss of pollinators would likely push many people below the nutritional threshold.^9^

“It’s important to note that this occurred mostly just for vitamin A in our study,” Ellis says. “For other nutrients, such as iron, pollinator declines may make no difference. It depends on if individuals are consuming foods that are highly dependent on pollinators and if they are getting most of their nutrients from those foods.”

The apparently negligible impact of pollinator declines on nutrition in countries where people are already very nutrient deficient could change if public health efforts bring the population to better health. “If other factors improved overall nutrition so that populations weren’t so malnourished, then the change from pollination might make a difference,” says Ricketts. Similarly, if the diets of well-nourished populations were to deteriorate for other reasons, then pollination changes might begin to matter for them as well.

Ricketts and his colleagues are continuing studies on how pollination affects human health, incorporating behavioral and dietary choices. For example, if pollinator populations were to decline, people who get their vitamin A from squash, which depends on insect pollination, may be able to switch to vitamin A–rich sweet potatoes, which have similar texture but don’t depend on insects. But would they?

“You can logic your way through it,” Ricketts says, “but we wouldn’t have predicted what we found based on just looking at these big global databases of food. … We’re finding that understanding human behavior is often really critical in figuring out whether nature helps human health.”

“The Ellis paper is a great example of what we need to do more of to understand the real vulnerability in the system,” says Chaplin-Kramer, who has begun a major project modeling how local nutrition might be affected by different agricultural interventions in Ghana and Burkina Faso. “The point is to connect more to the demand for the pollination service based on actual diet, rather than just the supply of the service,” she says.

Herren believes this emerging line of research is quite important. “We have spent far too long looking solely at calories as the answer to food security,” she says, “and not nutrition security.”
