# Erratum: “Pollinator Power: Nutrition Security Benefits of an Ecosystem Service”

**DOI:** 10.1289/ehp.123-A252

**Published:** 2015-10-01

Nicole W. 2015. Pollinator power: nutrition security benefits of an ecosystem service. Environ Health Perspect 123(8):A210–A215; http://dx.doi.org/10.1289/ehp.123-A210

This article was originally published under the incomplete title “Pollinator Power: Benefits of an Ecosystem Service.” *EHP* regrets the error.

